# Controlling the Stereodynamics of the F^–^ + NH_2_Cl S_N_2 Reaction by Vibrational Excitation: A Quasi-Classical Study

**DOI:** 10.1021/acsphyschemau.6c00092

**Published:** 2026-07-13

**Authors:** Deján Drágity, Gábor Czakó, Dóra Papp

**Affiliations:** † Department of Physical Chemistry and Materials Science, Institute of Chemistry, 37442 University of Szeged, Rerrich Béla tér 1, Szeged H-6720, Hungary; ‡ MTA-SZTE Lendület “Momentum” Computational Reaction Dynamics Research Group, Interdisciplinary Excellence Centre and Department of Physical Chemistry and Materials Science, Institute of Chemistry, 37442 University of Szeged, Rerrich Béla tér 1, Szeged H-6720, Hungary

**Keywords:** vibrational mode-specificity, N-centered S_N_2, stereoselectivity, multi-inversion, retention pathways, potential energy surface, quasi-classical trajectory

## Abstract

Here we report how specific reactant vibrational excitations impact the dynamics of the F^–^ + NH_2_Cl reaction, with a special emphasis on the stereoselectivity of its S_N_2 channel, by performing quasi-classical trajectory simulations on a high-quality ab initio analytical potential energy surface. Exciting the N–Cl stretching is found to enhance the S_N_2 reactivity the most, while N–H stretching excitation is the most efficient in promoting proton transfer. The stereospecificity of the title reaction can be drastically manipulated by exciting (1) the umbrella vibration, which facilitates repeated inversion through a H-bonded entrance channel minimum and transition state, thereby making multi-inversion the dominant mechanism over a wide collision energy range, accompanied by the complete loss of stereoselectivity; (2) the N–H stretching motion, which helps the H-bonded complex to form and thus lowers the inversion barrier around the N atom, thereby also promoting multi-inversion and racemization; and (3) the N–Cl stretching mode, which favors direct Walden inversion over complex-forming multi-inversion, resulting in reinforced stereoselectivity. The excess vibrational energy mainly flows into the internal degrees of freedom of the products, and while making the S_N_2 reaction more direct, it has an overall moderate impact on proton-transfer dynamics.

## Graphical abstract

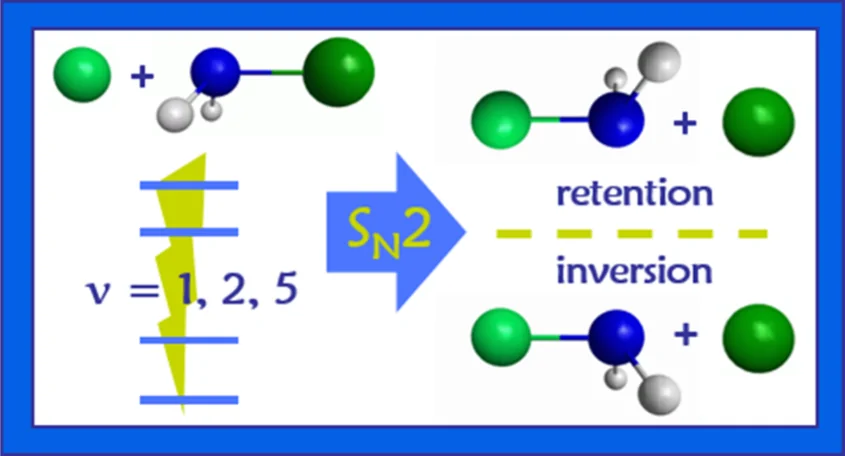

## I Introduction

Controlling chemical processes by selectively exciting various vibrational modes of the reactants has become a central topic in modern reaction dynamics studies. Vibrational mode-specific effects were first predicted by theory in the case of the H + H_2_O reaction in 1984.^1^ Later experiments confirmed the theoretical predictions,^2,3^ and mode-specific investigations were initiated for even larger systems, like the reactions of methane with different atoms.^4–12^ Besides the early experimental and theoretical studies on neutral systems,^1–12^ mode-specific effects were also investigated for ion–molecule reactions, such as the bimolecular nucleophilic substitution (S_N_2).^13–25^ These mode-specific studies on S_N_2 reactions always focused on carbon-centered systems, e.g., reactions of methyl- and ethyl-halides with different nucleophiles such as F^–^ and OH^–^, except for our very recent work on the F^–^ + SiH_3_Cl system.^25^

In the present study we plan to extend the scope of mode-specific ion–molecule chemistry by investigating a nitrogen-centered reaction, F^–^ + NH_2_Cl. S_N_2 reactions at nitrogen centers have a long history involving stationary-point characterizations, kinetics, and dynamics studies in both the gas phase and aqueous environments.^26–34^ In 2021 we developed a high-level analytical ab initio potential energy surface (PES) for the F^–^ + NH_2_Cl reaction.^32^ Quasi-classical trajectory (QCT) simulations on this PES revealed a multi-inversion mechanism, which undermined stereoselectivity and resulted in significant reactivity with retention of the initial configuration.^32^ In 2025 other unconventional S_N_2 pathways were revealed at nitrogen centers, like roundabout and hydride transfer.^34^ Motivated by the recent interest in nitrogen-centered S_N_2 reactions and the lack of mode-specific investigations for these systems, we plan to report a detailed QCT study on the F^–^ + NH_2_Cl reaction where trajectories are initiated from specific vibrational states of the reactant molecule. The simulations utilize our analytical PES,^32^ which describes both the S_N_2 channel leading to Cl^–^ + NH_2_F and the proton-abstraction pathway producing HF + NHCl^–^. The two competing reactions are different because S_N_2 is an exothermic barrier-less process, whereas proton transfer is endothermic; therefore, some energy has to be invested into the system to reach the products. It will be interesting to find out how specific vibrational excitations of the reactant affect the competition of these dynamically different channels. Furthermore, a central question in S_N_2 reactions is how stereospecificity is maintained. As mentioned above, a proton-abstraction-induced multi-inversion of the reactant molecule can undermine stereoselectivity, especially at low collision energies,^32^ and the racemization effect may become more pronounced if specific vibrational modes of the NH_2_Cl molecule are initially excited. These questions along with others related to, for example, energy transfer, will be answered by the present simulations.

In Section 2, we describe the computational details of the mode-specific trajectory simulations. The results are presented and discussed in Section 3, and the paper ends with a summary and conclusions in Section 4.

## II Computational Details

Vibrational mode-specific QCT simulations are carried out on a full-dimensional analytical PES,^32^ previously developed with the Robosurfer program package^35^ for the F^–^ + NH_2_Cl reaction, fitted on 16 780 CCSD­(T)-F12b/aug-cc-pVTZ^36,37^ energy points with permutationally invariant polynomials^38^ as the basis. The least-squares fit uses 4285 coefficients, and the highest fitting order is 6. The PES has a root-mean-square error of 0.56 kcal/mol regarding the 63 kcal/mol energy range above the global minimum.

The quasi-classical trajectories are initiated either from the ground state or from a vibrationally excited state of the NH_2_Cl reactant molecule. Zero-point energy (ZPE) and the vibrational excitation energies are set by using standard normal-mode sampling.^39^ The fundamental harmonic energies in kcal/mol determined on the PES are as follows for the normal vibrations of NH_2_Cl: 1.97 for N–Cl stretching, 3.16 for umbrella inversion motion (NH_2_ wagging), 3.46 for NH_2_ twisting, 4.70 for NH_2_ bending, 9.90 for symmetric NH_2_ stretching, and 10.15 for asymmetric NH_2_ stretching. All normal modes are given a one-quantum excitation, and since the N–Cl stretching and umbrella motions correspond directly to the reaction coordinate of the S_N_2 reaction, we further excite these modes with two and five quanta, as well. Higher excitations of the umbrella vibration are also interesting due to the crucial role of this motion in the stereodynamics of the reaction. In one simulation only one normal vibration is excited by the respective number of quanta. Our previous studies of polyatomic reactions,^20,40,41^ including the analogous carbon-centered F^–^ + CHD_2_Cl system,^40^ have shown that intramolecular vibrational redistribution (IVR) may occur during the QCT simulations; however, the initially excited modes preserve their mode-specific character until the reactive encounter.

The reactants have random orientation at the beginning of the trajectories, and the simulation is propagated using a 0.0726 fs time step. The trajectories finish when the largest final interatomic distance becomes 1 bohr larger than the largest initial one. The initial distance between the center of mass of NH_2_Cl and the F^–^ ion is $\sqrt{x^{2}+b^{2}}$ , where *x* = 30 bohr and the impact parameter *b* is varied between 0 and *b*_max_ (where the reaction probability becomes zero) with a step size of 1 bohr. The trajectories are run at five different collision energies: 0.9, 10, 20, 30, and 40 kcal/mol. 5000 simulations are carried out at each collision energy–*b*–excitation combination.

Integral cross sections (ICSs) for the S_N_2 and proton-transfer (PT) reaction channels are calculated by a *b*-weighted numerical integration of the *P*(*b*) reaction probabilities. For the PT reaction we also calculate various ZPE-constrained ICSs: “Soft”—the sum of the classical vibrational energies of the products at the end of the simulation must be larger than the sum of their ZPEs; “Hard”—this constraint is set separately for each product; and “NHCl^–^-ZPE”—the restriction is defined only for the polyatomic product. We also determine scattering angles for the reaction by binning the cosine of the included angle of the relative velocity vectors of the reactants and the products into 10 equidistant bins, where 180° signals backward scattering. We distinguish between inversion and retention in the case of the S_N_2 reaction by calculating the scalar products of the normal vector of the H–N–H plane with the N–F vector of the NH_2_F molecule and with the N–Cl vector of the NH_2_Cl reactant. If these scalar products have the same sign, the configuration is retained in the reaction; otherwise, inversion took place.

## III Results and Discussion

The schematic potential energy surface of the F^–^ + NH_2_Cl reaction is shown in Figure 1. As mentioned in Section 1, there are two competing channels: the exothermic S_N_2 and the endothermic proton transfer leading to the Cl^–^ + NH_2_F and HF + NHCl^–^ products, respectively. Both channels have submerged transition states (TSs), with very similar energies; considering zero-point vibration, the Walden inversion TS is below the PT TS by only 0.5 kcal/mol. The Walden inversion process goes through a central barrier separating deep hydrogen-bonded pre- and postreaction minima, whereas the PT TS is rather product-like and the postreaction complex is just slightly below the TS. Inversion of the reactant and S_N_2 product can proceed via relatively low-energy *C*_2*v*_ TSs, and this inversion barrier can be further reduced by F–H-bonding in the entrance channel. As our previous dynamics simulations showed, this prereaction H-abstraction mediated inversion, which often occurs multiple times, is responsible for the undermined stereospecificity of the F^–^ + NH_2_Cl S_N_2 reaction. The front-side attack pathway, which also provides S_N_2 products with retention of the initial configuration, has a high barrier and therefore plays a minor role in the dynamics of the title reaction.

**Figure 1:**
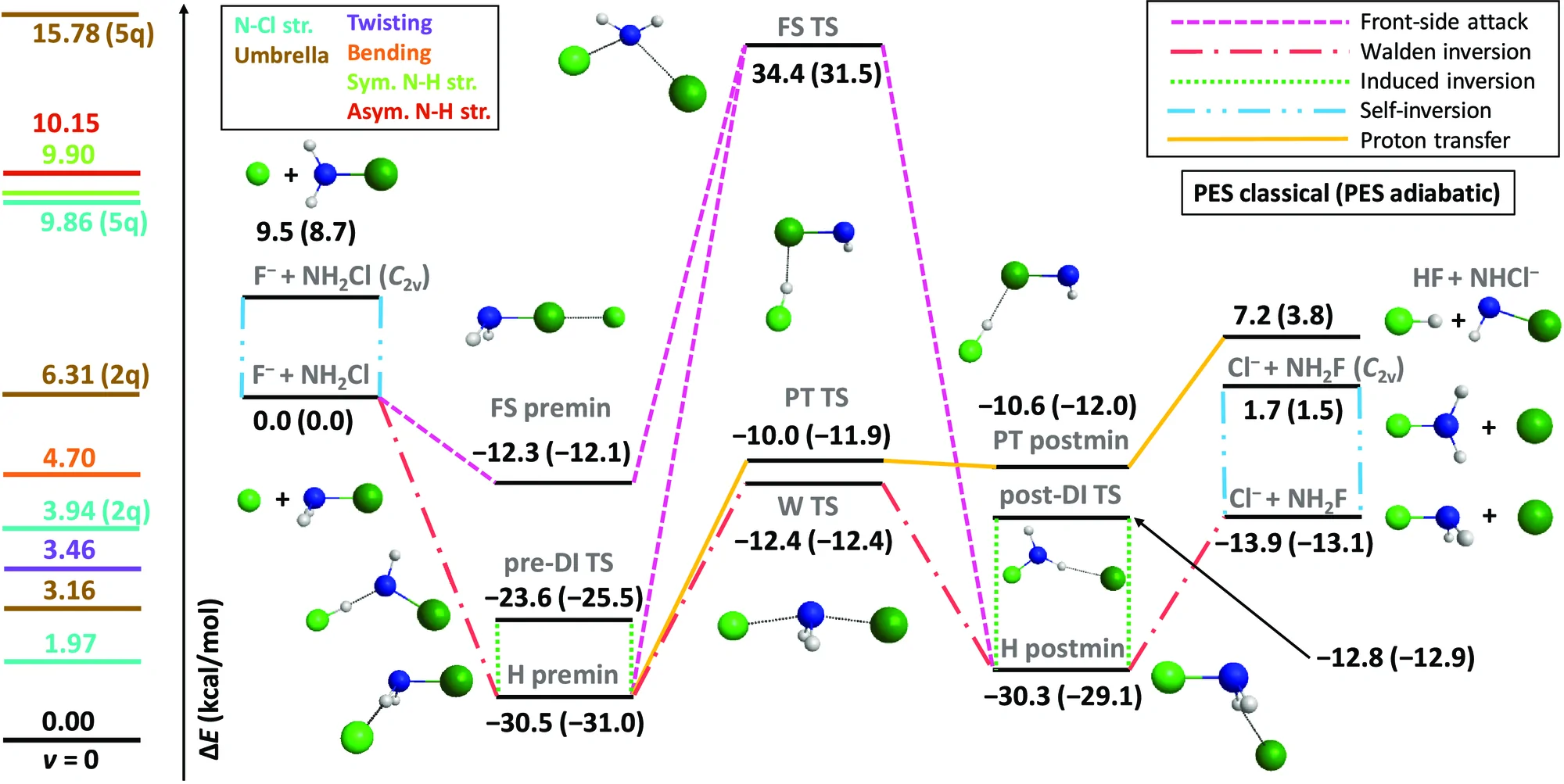
Schematic potential energy surface of the F^–^ + NH_2_Cl reaction showing classical and adiabatic relative energies of the stationary points obtained on the PES^32^ along with reactant harmonic vibrational energy levels, with N–Cl stretching and umbrella vibrations excited with two (2q) and five (5q) quanta. Adapted with permission from ref 32. Copyright 2021 Royal Society of Chemistry.

In Figure 2, we present the integral cross sections of the S_N_2 and PT channels as a function of the collision energy. S_N_2 ICSs divided into retention and inversion are shown in Figure S1. All ICSs decrease with increasing collision energy, which is not surprising for the barrierless S_N_2 reaction, where interaction time is the main promoting effect. However, even for the nonconstrained PT ICS, it is unusual and probably caused by the almost negligible endothermicity of the channel. Cross sections of the PT reaction are about three times larger than those of the S_N_2 reactions; however, only ZPE-violating trajectories contribute to the highest ICS values at the lowest collision energy of the PT reaction. In Figure 3, the ratio of the vibrationally excited and ground-state S_N_2 ICSs can be seen for the different normal modes as a function of collision energy, while Figure 4 shows the same quantities divided based on the configuration of the NH_2_F product. The ratio between retention and inversion ICSs for the various excitations is presented in Figure 5. PT ICS ratios (excited/ground) are depicted in Figure 6 as a function of collision energy, while PT reactivity enhancements obtained with different ZPE constraints (see Section 2) are plotted in Figures S2 and S3.

**Figure 2:**
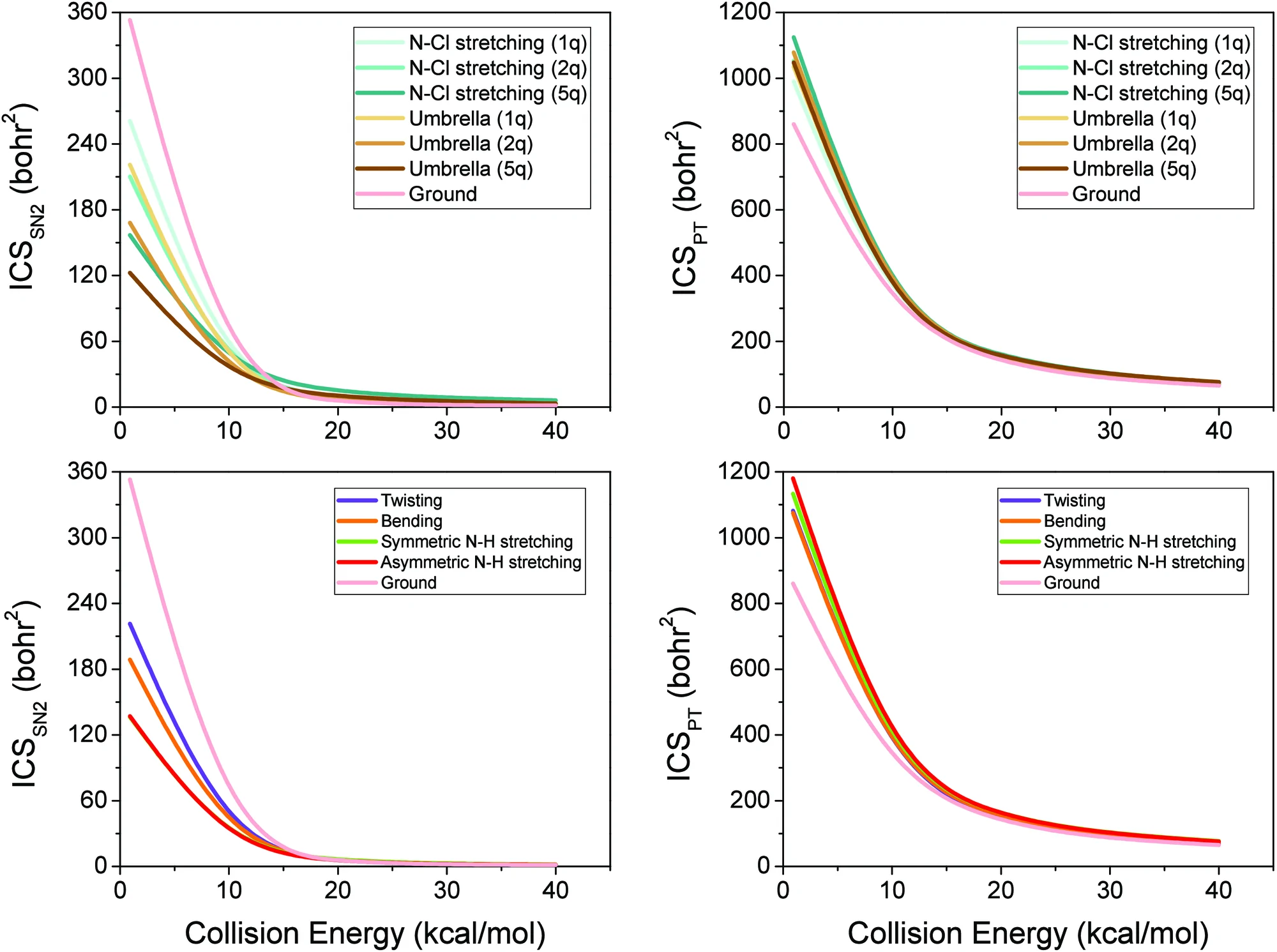
Integral cross sections of the S_N_2 (first column) and proton-transfer (without ZPE constraints, second column) channels of the F^–^ + NH_2_Cl reaction for various excitations of the reactant vibrational normal modes and for the unexcited reaction (“Ground”) as a function of collision energy.

**Figure 3:**
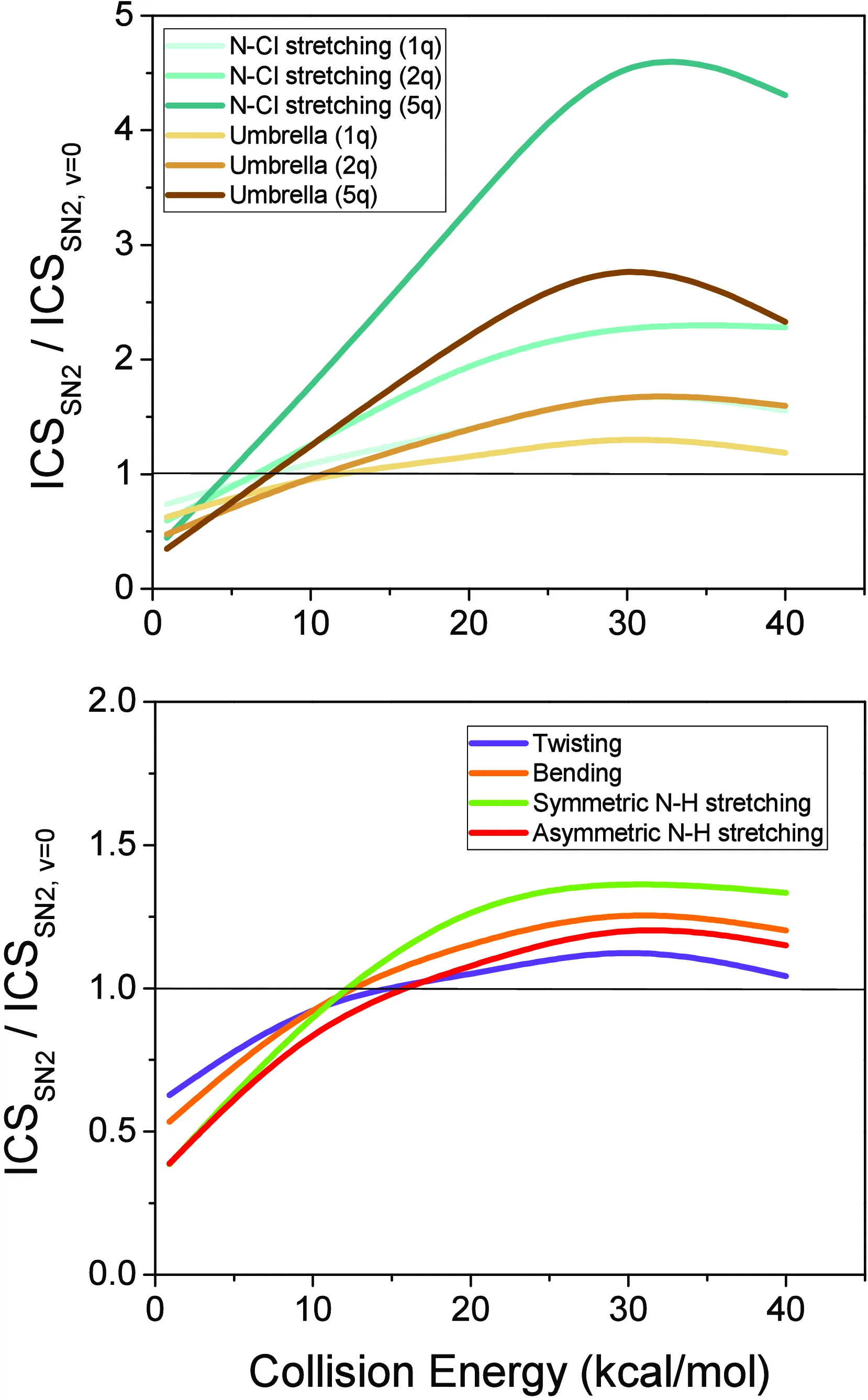
Ratios of the integral cross sections of the vibrationally excited and the nonexcited (*v* = 0) F^–^ + NH_2_Cl → Cl^–^ + NH_2_F S_N_2 reaction for various excitations of the reactant vibrational normal modes as a function of collision energy.

**Figure 4:**
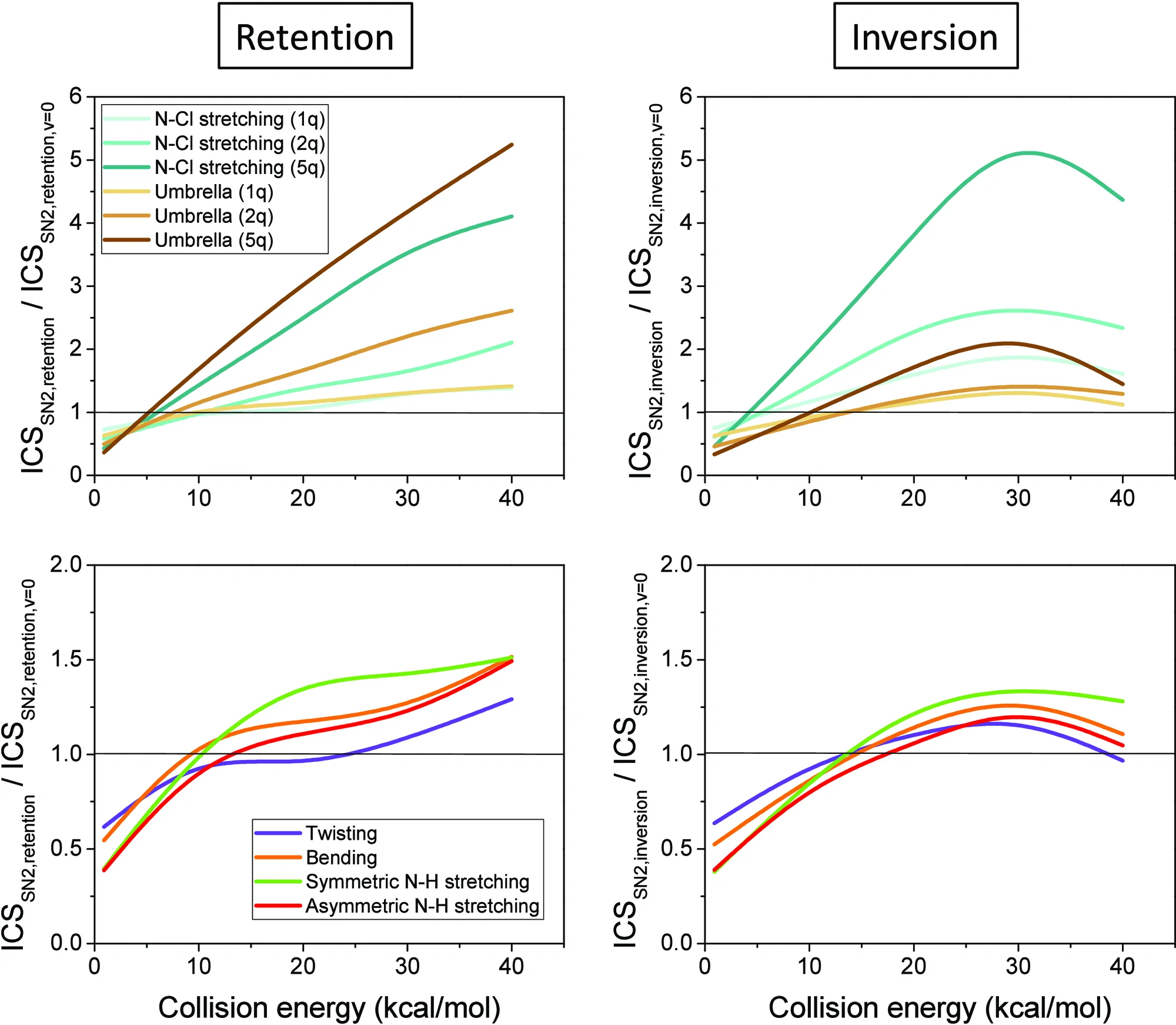
Ratios of the integral cross sections of the vibrationally excited and the nonexcited (*v* = 0) F^–^ + NH_2_Cl → Cl^–^ + NH_2_F S_N_2 reaction divided based on the configuration of the NH_2_F product relative to the NH_2_Cl reactant into inversion and retention for various excitations of the reactant vibrational normal modes as a function of collision energy.

**Figure 5:**
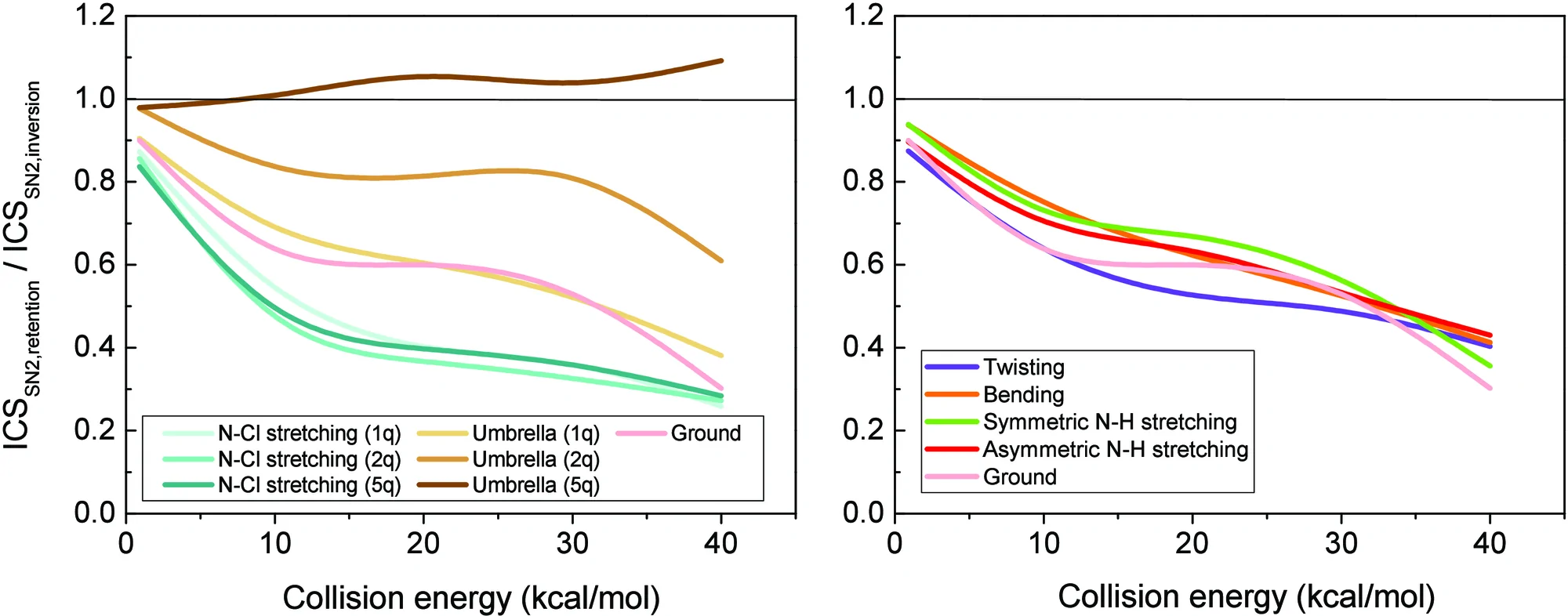
Ratios of the retention and inversion integral cross sections of the F^–^ + NH_2_Cl → Cl^–^ + NH_2_F S_N_2 reaction for various excitations of the reactant vibrational normal modes and for the unexcited reaction (“Ground”) as a function of collision energy.

**Figure 6:**
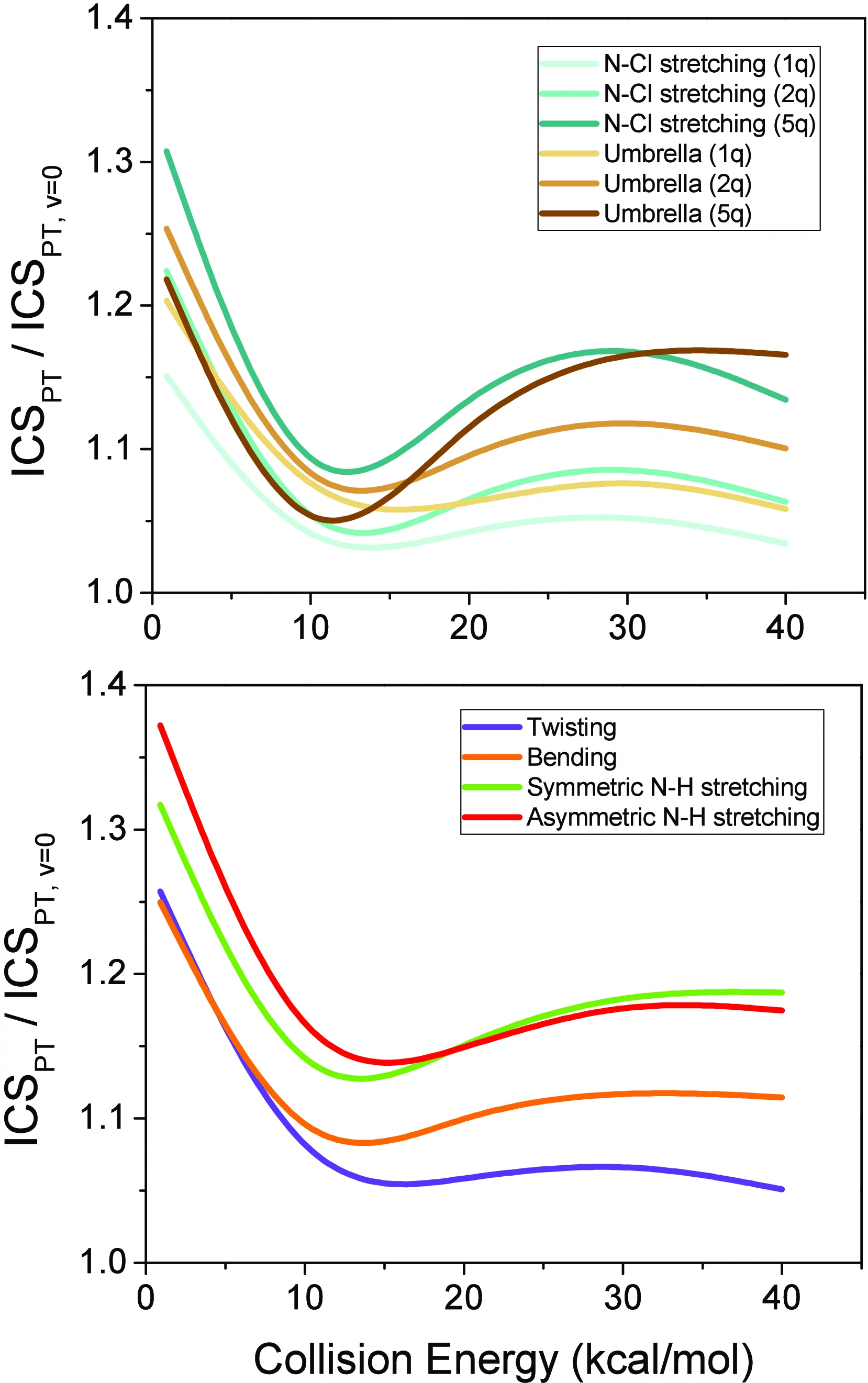
Ratios of the integral cross sections of the vibrationally excited and the nonexcited (*v* = 0) F^–^ + NH_2_Cl → HF + NHCl^–^ proton-transfer reaction for various excitations of the reactant vibrational normal modes as a function of collision energy.

It is clear that promotion of the S_N_2 channel happens only above a collision energy of 10 kcal/mol (a decrease in reactivity by approximately half is observed at 0.9 kcal/mol), whereas proton-transfer reactivity is significantly increased at low energies upon vibrational excitation, probably at the expense of the S_N_2 reaction. The unconstrained PT ICSs in Figure 2 show unrealistic reactivity for the ground-state and the lowest-energy N–Cl stretching-excited PT reactions (with an endothermicity of 3.8 kcal/mol) at the lowest collision energy, which is eliminated using the “Soft” and “Hard” ZPE constraints (see below). In the middle energy range, promotion of the PT channel falls back, while that of S_N_2 increases, thus leveling the competition between the two reaction paths. At the highest energy, the vibrational enhancement drops for both reaction channels, presumably as a result of the faster approach of the reactants that prevents vibrational motion to take effect.

The different ZPE constraints set for the PT ICSs have quite unrealistic effects, as vibrational excitation overcompensates ZPE violation, which is more relevant for PT, owing to its endothermicity, than for S_N_2 (see Figures S2 and S3). At the lowest collision energy, ratios are missing when the “Soft” and “Hard” restrictions are applied because ground-state (and the one-quantum N–Cl-excited) reactivity vanishes, being consistent with the endothermicity of PT. When restricting only the ZPE of NHCl^–^, where the ground-state reaction still has a small reactivity, a large enhancement appears at this energy. At higher energies, the “Hard” constraint shows the largest, overestimated enhancements, especially when the N–H stretching mode is excited; the “NHCl^–^” and the “Soft” constraints exhibit only moderate vibrational compensation. Every ZPE restriction changes the shape of the curves to decrease monotonically, which suggests that ZPE violation is mainly responsible for the alternating vibrational enhancement of the PT channel.

As for mode-specificity, we can see that the impacts of all vibrational modes fall in the same magnitude when excited with one quantum in the case of the S_N_2 reaction. The increased excitation of the umbrella and the N–Cl stretching modes with two and five quanta is accompanied by a gradual increase of their effects. Excitation of these two modes starts to enhance S_N_2 reactivity at a collision energy of 10 kcal/mol, whereas exciting the other modes increases reactivity only from 20 kcal/mol. Interestingly, from the two most relevant modes for S_N_2, the N–Cl stretching vibration, despite carrying less excitation energy than the umbrella mode, promotes the reaction more effectively, reaching nearly a factor of 5 of enhancement. However, the umbrella excitation also has a significant effect, almost a maximum of a 3-fold increase in reactivity at a collision energy of 30 kcal/mol.

One-quantum excitations of the twisting, bending, symmetric, and asymmetric N–H stretching modes make the ICSs decrease by half at the lowest collision energy, while reaching a 5–40% enhancement at the three highest energies. Among these modes, the most effective in promoting the S_N_2 reaction (even more so than the one-quantum umbrella excitation and comparable with the one-quantum N–Cl one) is the symmetric N–H stretching. Interestingly, this mode is the most efficient in inhibiting the reaction at the lowest collision energy as well. Asymmetric N–H stretching excitation has a minor effect, and twisting is less efficient both in hindering and in promoting the reaction at the respective energies.

The reactivity enhancements, plotted in Figure 4, show a similar shape for inversion as for the overall S_N_2 process, especially at higher energies, where direct Walden inversion usually dominates the reaction over multi-inversion, due to the decreasing interaction time. The only exception is the umbrella vibration, the promoting effect of which decreases significantly for inversion and increases for retention. Enhancements corresponding to the retained configuration show a steep increase as collision energy increases for the N–Cl stretching and umbrella excitations, while the curves just slightly rise for the remaining normal modes. Dropping ratios at the highest energy are observed only for the inversion curves, probably because the retention of configuration is mainly the result of the less direct complex-forming multi-inversion mechanism; however, it may be complemented by front-side attack at this energy (of course, inversion may also come from multi-inversion).

Inspecting the left panel of Figure 5, it is evident that the umbrella excitation has the most pronounced impact on stereoselectivity, as expected. While the one-quantum excitation has only a minor effect, the two- and five-quantum excitations increasingly flatten the ratio curves, guiding the reaction more and more toward racemization. This means a much higher increase in the retention ratio at higher energies compared to the lower ones, consistent with Figure 4. The five-quantum umbrella excitation practically results in a fully racemic outcome of the S_N_2 reaction at all studied energies. This effect is a result of the promotion of the multi-inversion mechanism by pouring extra energy into the inversional motion around the N atom. In contrast to the ground-state process, in which multi-inversion dominates the S_N_2 reaction at only low collision energies, it turns out that high excitation of the umbrella vibration makes multi-inversion the primary reaction route of the S_N_2 channel throughout a wide energy range. Interestingly, the N–Cl stretching excitation also causes a major change in the stereodynamics of the S_N_2 reaction. Extra energy in this vibration elongates the N–Cl bond, which favors the direct Walden inversion process over the multi-inversion mechanism, resulting in a decreased retention ratio and thus a more stereoselective S_N_2 reaction. As collision energy rises, N–Cl excitation loses some of its impact because Walden inversion is already the dominant pathway at higher energies. Although the retention/inversion ICS ratios exhibit larger uncertainties at the highest collision energies, a statistical test at the highest energy showed that using only half of the trajectories changes the ratios by about 10% on average without altering the overall conclusions.

Compared to the ground-state reaction, symmetric and asymmetric N–H stretching excitations also shift the stereodynamics toward racemization, even more than the one-quantum umbrella excitation, especially at lower collision energies, which inherently favor complex formation. Elongating the N–H bonds helps the H···F H-bond to form, thereby lowering the inversion barrier around the central N atom and promoting multi-inversion. Symmetric N–H stretching has a slightly larger effect in increasing the retention ratio. Exciting the bending of the NH_2_ group appears to help configuration retention at lower collision energies, whereas the twisting excitation prevents it in the middle and promotes it in the high energy range, with respect to the unexcited reaction.

Excitation of N–H stretching is found not only to promote racemization in the S_N_2 reaction via facilitating the H-bonded complex formation; it also has the strongest promoting effect on the PT channel, as seen in Figure 6. It increases PT reactivity by 15–35%, with the impact of the asymmetric N–H excitation slightly exceeding that of the symmetric one. NH_2_ bending and twisting cause a more minor, but still relevant, enhancement of around 5–25%, with the bending excitation being more intense. The excitation of N–H stretching seems to promote proton transfer in a similar order of magnitude as how it enhances the S_N_2 reactivity, since in the former case this vibration coincides directly with the reaction coordinate, while in the latter this excitation facilitates the formation of the F–H bond in H premin, which reduces the inversion barrier and thus favors racemization in S_N_2.

As expected, exciting the N–Cl stretching and the umbrella vibrations, even with multiple quanta, is less effective in enhancing the PT reactivity than the excitation of the N–H stretching modes, and it acts less effectively on PT than on S_N_2, being far less coupled to the PT reaction coordinate. These excitations show a deeper “minimum” in the ratio curves, consistent with the sharper increase in the same curves for S_N_2 in Figure 3. Interestingly, in the case of the PT channel, none of the excited vibrations hinder the reaction.

Opacity functions, i.e., the reaction probabilities as a function of the impact parameter *b*, of the S_N_2 channel at the three lowest collision energies, where the S_N_2 reactivity is the most relevant, for different reactant excitations are shown in Figure 7. These quantities at the two highest energies are also plotted in Figure S4. The *b*_max_ values, which decrease greatly (approximately halving) above the lowest collision energy, are not much affected by reactant vibrational excitations; however, the reactive *b*-range is somewhat extended as the excess vibrational energy increases. At a collision energy of 0.9 kcal/mol, probabilities of the vibrationally excited reactions drop considerably with respect to the ground-state reaction, especially at small *b* values, being consistent with the ratios shown in Figure 2. In contrast, as collision energy is increased, the vibrationally excited S_N_2 probabilities rise, specifically at large *b* values, implying that vibrational excitation might promote the stripping mechanism, favored at large *b* values. This shift to large impact parameters is more and more visible with increasing collision energy.

**Figure 7:**
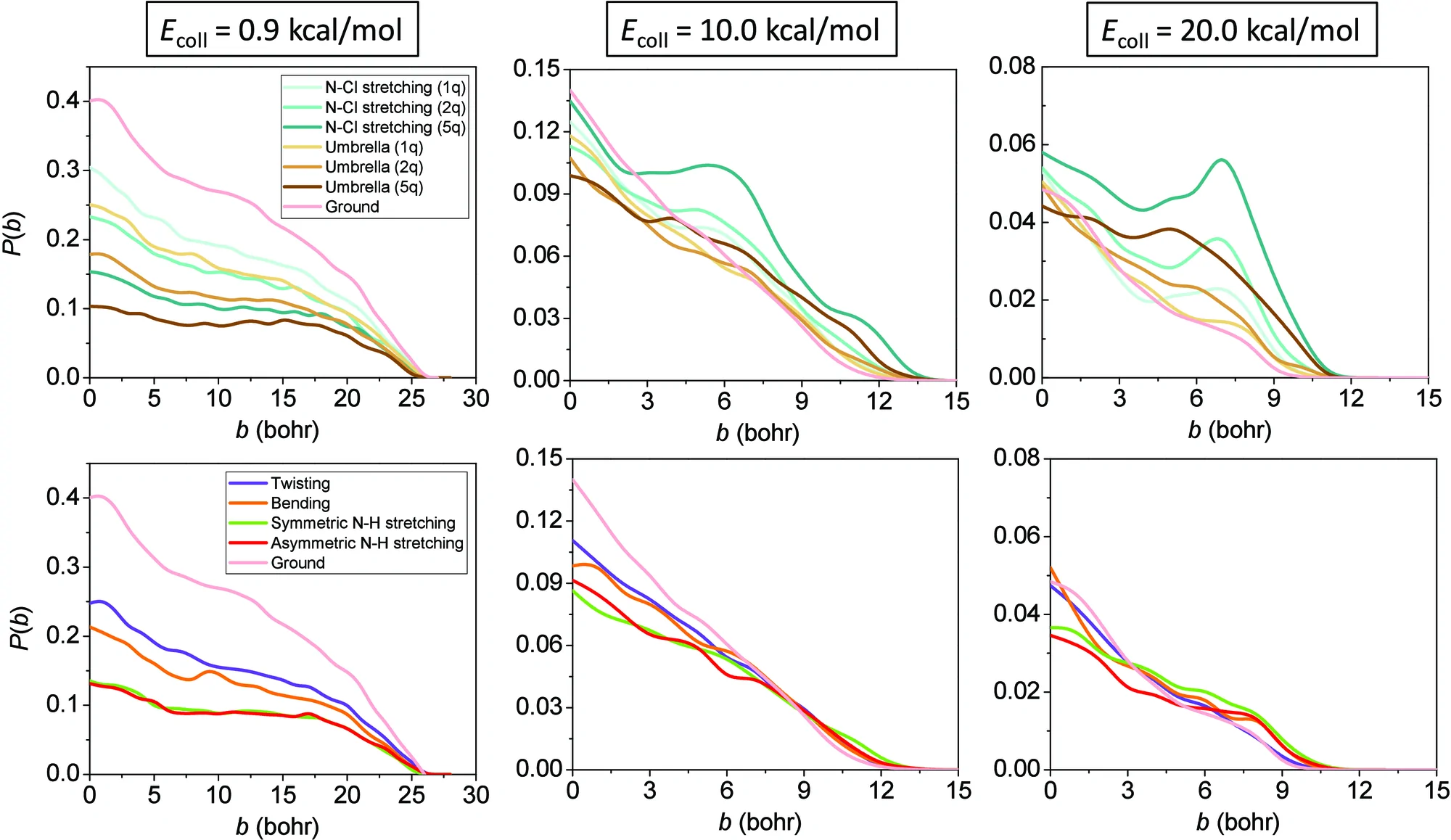
Opacity functions (reaction probabilities *P* as a function of the impact parameter *b*) of the F^–^ + NH_2_Cl → Cl^–^ + NH_2_F S_N_2 reaction for various excitations of the reactant vibrational normal modes and for the unexcited reaction (“Ground”) at different collision energies.

In Figure S5, we plot the scattering angle distributions corresponding to the S_N_2 channel of the title reaction. Besides a slight increase in preference for forward stripping with increasing collision energy for every reaction, mode-specificity is hardly observed in the distributions, which is not surprising as the *b*_max_ values are not much affected by vibrational excitation. Only the curves at a collision energy of 20 kcal/mol show forward preference in the case of the higher N–Cl stretching excitations, in accordance with the large *b* maxima of the opacity functions in Figure 7. Both the opacity functions and the scattering angle distributions at the two highest collision energies reflect statistical uncertainty because of the low reactivity of the S_N_2 channel in these cases.

To investigate how vibrational excitation impacts the energy flow in the S_N_2 reaction and thus its directness, we present the internal energy distributions of the NH_2_F product in Figure 8 and the relative translational energy distributions of the products in Figure 9, for the three lowest collision energies. The distributions at the two highest energies can be seen in Figures S6 and S7. As seen from the ground-state internal energy distributions, the S_N_2 reaction is rather indirect at the three lowest collision energies, as the curves peak at the maximal available energy, indicating high-internal-energy complex formation. At the lowest collision energy, where reactant vibrational excitations hinder the reaction, no change is visible in directness; however, the blue shift of the maxima suggests that vibrational excitation remains mainly in the internal degrees of freedom of NH_2_F. At higher collision energies, where reactivity is increased by vibrational excitation, the excited internal energy curves widen, and their maxima are relocated toward lower energies, reflecting more and more direct dynamics. The largest effects are found in the case of the five-quantum umbrella excitation, indicating vibrationally highly excited products and accelerated complex formation. Five-quantum N–Cl stretching, two-quantum umbrella, and N–H stretching excitations have a significant impact, as well. Basically, no ZPE violation is seen in the exothermic S_N_2 reaction. Consistent with these results, the translational energy curves show only minor mode-specificity and generally exhibit colder distributions, which turn somewhat hotter upon excitation. At the highest collision energies, excess reactant vibrational energy seems to flow less effectively into product internal energy.

**Figure 8:**
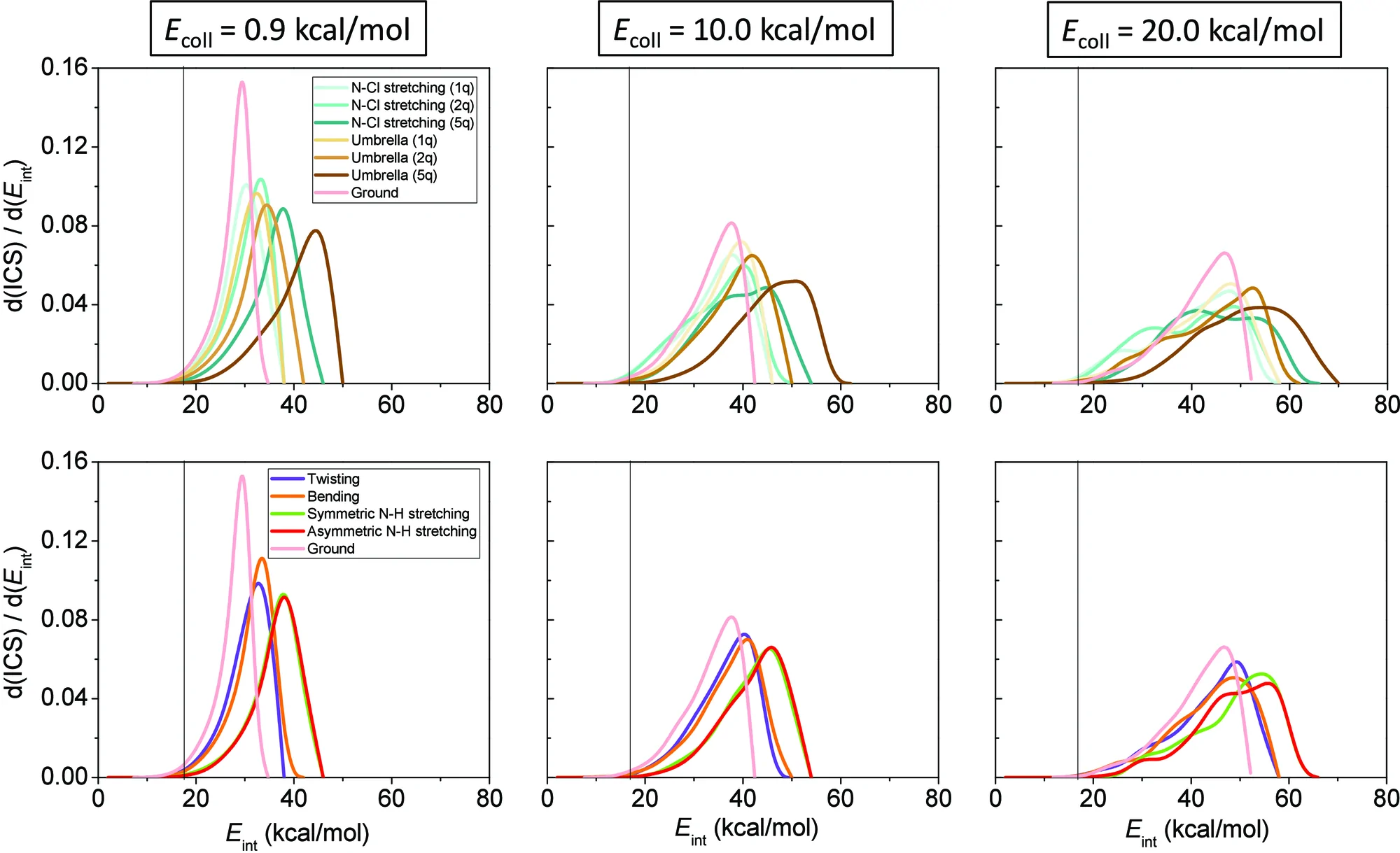
Internal energy distributions of the NH_2_F product of the F^–^ + NH_2_Cl → Cl^–^ + NH_2_F S_N_2 reaction for various excitations of the reactant vibrational normal modes and for the unexcited reaction (“Ground”) at different collision energies. The vertical line denotes the ZPE of the NH_2_F product.

**Figure 9:**
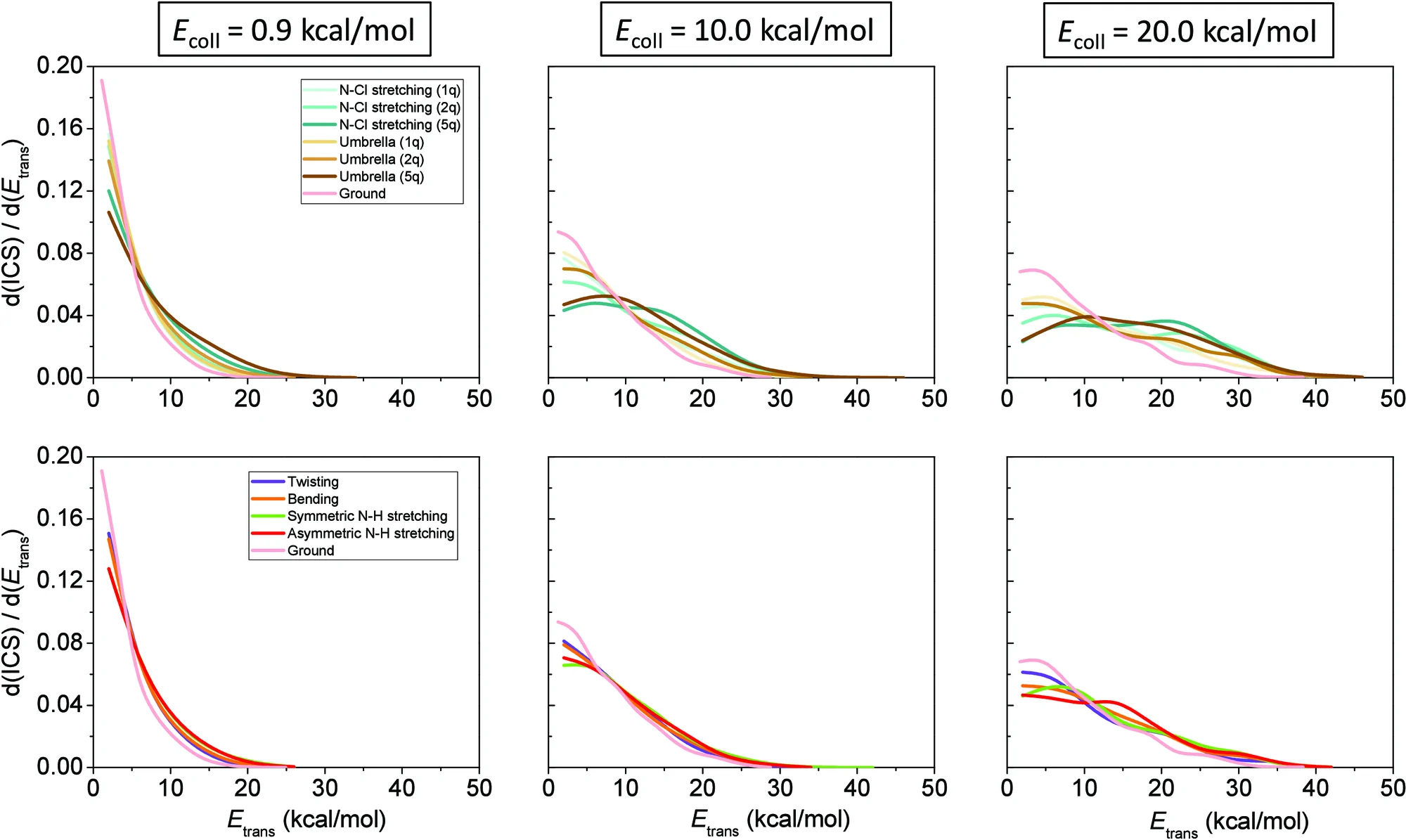
Relative translational energy distributions of the products of the F^–^ + NH_2_Cl → Cl^–^ + NH_2_F S_N_2 reaction for various excitations of the reactant vibrational normal modes and for the unexcited reaction (“Ground”) at different collision energies.

We also explore the dynamics of proton transfer, which is a fairly competitive route with the S_N_2 pathway. Unrestricted (i.e., involving no ZPE constraint) opacity functions, scattering angle distributions, and internal and translational energy distributions are shown in Figures S8–S11, respectively, for this channel. Reaction probabilities are not affected significantly by reactant vibrational excitation, except at the lowest collision energy, where excess vibrational energy allows the endothermicity of the reaction path to be overcome. Not surprisingly, the N–H stretching vibrations have the strongest effects, closely followed by the high-quantum N–Cl and umbrella excitations. Scattering angles are practically unaffected by vibrational excitation, and only the highest excited umbrella mode causes a slight forward preference. In contrast, internal energy distributions of NHCl^–^ feature a clear mode-specificity as excess vibrational energy visibly flows into the internal motion of the polyatomic product indicated by widening curves and blue-shifting maxima with increasing excitation energy. In line with this, only a small amount of the vibrational energy is converted into product recoil in the PT reaction, resulting in minor excitation effects in the translational energy distributions. With increasing collision energy, mode-specificity eventually vanishes, as can be observed in all the dynamic quantities corresponding to proton transfer.

## IV Summary and Conclusions

Here we explore the impact of reactant vibrational excitation on the dynamics of the F^–^ + NH_2_Cl reaction, with a special focus on the stereodynamics of its S_N_2 pathway. For this we perform quasi-classical trajectory simulations on a recently developed high-quality ab initio analytical PES by exciting all the normal vibrational modes of NH_2_Cl, the N–Cl stretching and the umbrella vibrations even with two and five quanta, at different collision energies. It turns out that at low collision energies, vibrational excitation hinders the S_N_2 channel while promoting proton transfer. At higher energies, eventually all vibrational excitations result in increased reactivity of both channels. The effect of the N–H/N–Cl stretching and umbrella motions excited by one quantum is comparable for the S_N_2 reaction, and with increasing vibrational excitation of the latter two, reactivity enhancement is usually more and more pronounced. N–Cl stretching is the most efficient in increasing S_N_2 reactivity, whereas N–H stretching has the strongest impact on the PT reaction. Bending and twisting modes have a moderate impact on the reactivity of the title reaction. The main finding of the present work is that exciting specific vibrational modes allows for enhancing or quenching the stereoselectivity of the S_N_2 reaction: (1) Increasing umbrella excitation leads to the complete loss of stereospecificity with increasing collision energy by facilitating repeated inversion through a H-bonded entrance-channel minimum, thereby making multi-inversion, which is the prevalent mechanism at low collision energies in the ground-state reaction, the dominant pathway in a wide energy range. (2) N–H stretching excitation also shifts the S_N_2 reaction toward racemization by helping the H-bonded complex to form, thus lowering the inversion barrier around the central N atom and consequently promoting multi-inversion. (3) Exciting the N–Cl stretching, however, supports stereoselectivity by favoring the direct Walden inversion pathway over complex-forming multi-inversion. In both the S_N_2 and PT reaction, the excess vibrational energy flows more efficiently into the internal degrees of freedom of the products than into product recoil. While vibrational excitation makes the S_N_2 channel more direct with increasing collision energy, it has an overall minor effect on PT dynamics.

## Supplementary Material



## Notes

Integral cross sections of the S_N_2 channel divided based on retention and inversion as a function of collision energy; ratios of the integral cross sections of the vibrationally excited and the nonexcited PT reactions obtained with different ZPE constraints as a function of collision energy; scattering angle distributions for the S_N_2 channel at all collision energies; S_N_2 opacity functions as well as internal and translational energy distributions at the two highest collision energies; and opacity functions, scattering angle distributions, and internal and translational energy distributions of the PT channel at all collision energies (PDF)
