# Supplementary material for: Controlling the Stereodynamics of the F– + NH2Cl SN2 Reaction by Vibrational Excitation: A Quasi-Classical Study
Source: ACS Phys Chem Au. 2026 Jul 13;6(5):1299–307. doi: 10.1021/acsphyschemau.6c00092 (PMC13614146; doi:10.1021/acsphyschemau.6c00092)
Supplement: Supplementary file 1 [file pg6c00092_si_001.pdf]

Supporting Information

Controlling the Stereodynamics of the F^{-} + NH_2Cl S_N2 Reaction by Vibrational Excitation: A Quasi-Classical Study

Deján Drágity,[a] Gábor Czakó,[b] and Dóra Papp[a,*]

[a] Department of Physical Chemistry and Materials Science, Institute of Chemistry, University of Szeged, Rerrich Béla tér 1, Szeged H-6720, Hungary

[b] MTA-SZTE Lendület "Momentum" Computational Reaction Dynamics Research Group, Interdisciplinary Excellence Centre and Department of Physical Chemistry and Materials Science, Institute of Chemistry, University of Szeged, Rerrich Béla tér 1, Szeged H-6720, Hungary

* E-mail: dorapapp@chem.u-szeged.hu

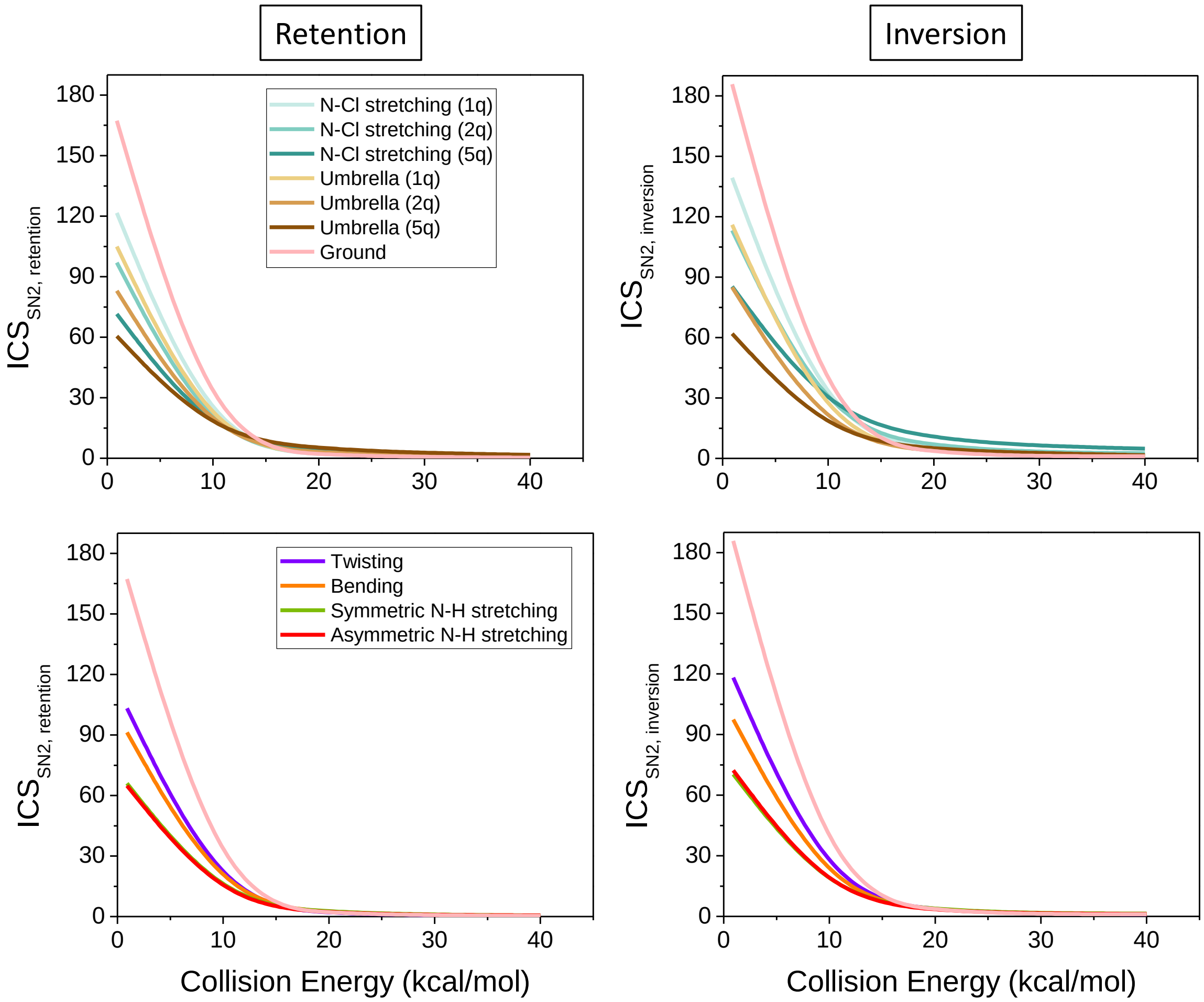

Figure S1. Integral cross sections of the S_N2 channel of the F^{-} + NH_2Cl reaction divided based on the configuration of the NH_2F product relative to the NH_2Cl reactant into inversion and retention in case of various excitations of the reactant vibrational normal modes and for the unexcited reaction (Ground) as a function of collision energy.

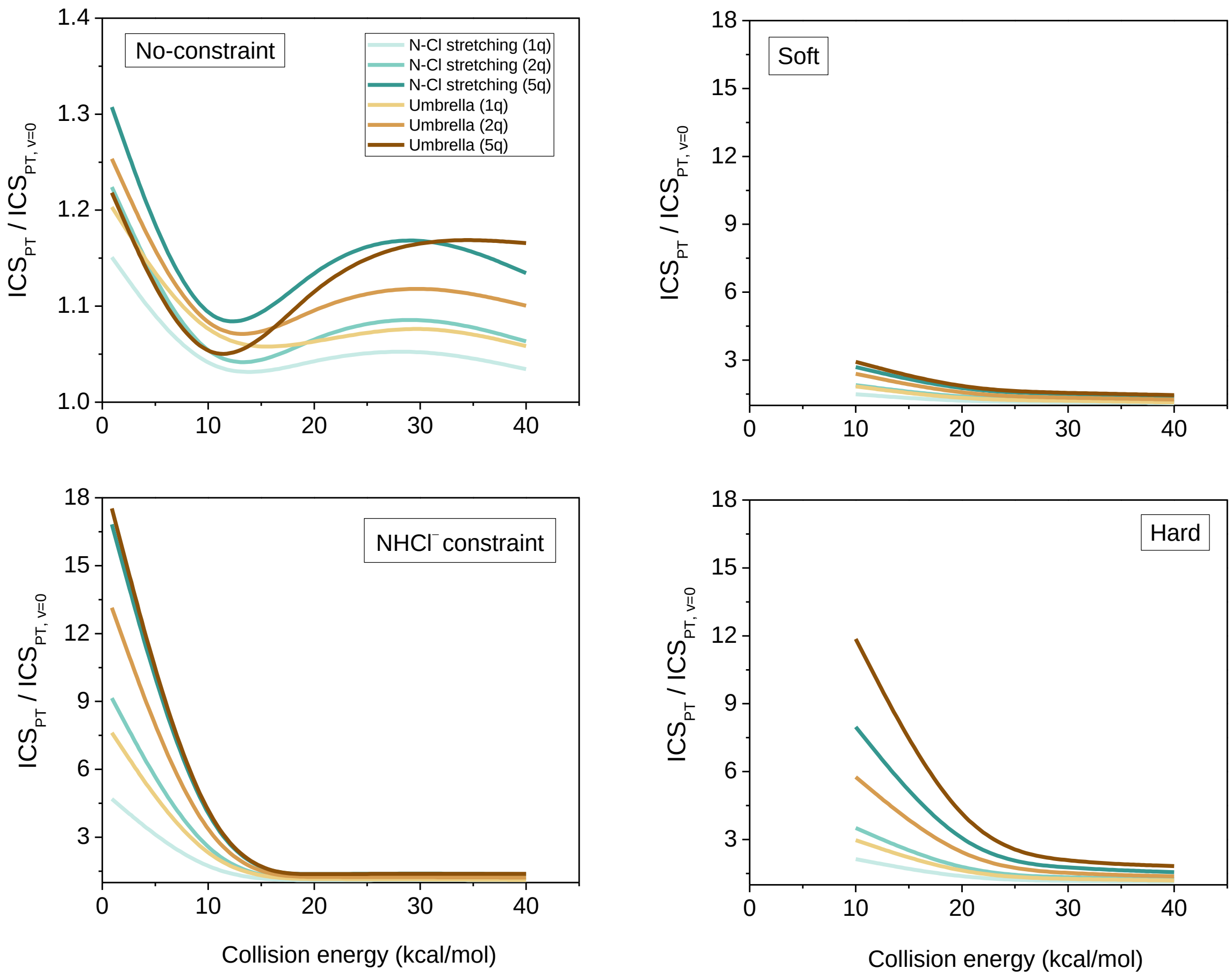

Figure S2. Ratios of the integral cross sections of the vibrationally excited and the non-excited (v = 0) proton-transfer channel of the F^{-} + NH_2Cl reaction in case of various excitations of the reactant vibrational normal modes (N-Cl stretching and umbrella modes excited by 1, 2, and 5 quanta) as a function of collision energy applying different zero-point-energy (ZPE) constraints: Soft - the sum of the vibrational energies of the products must be larger than the sum of the ZPEs, Hard - this restriction is set for each product separately, $NHCl^{-}$ constraint - the constraint is set only for $NHCl^{-}$.

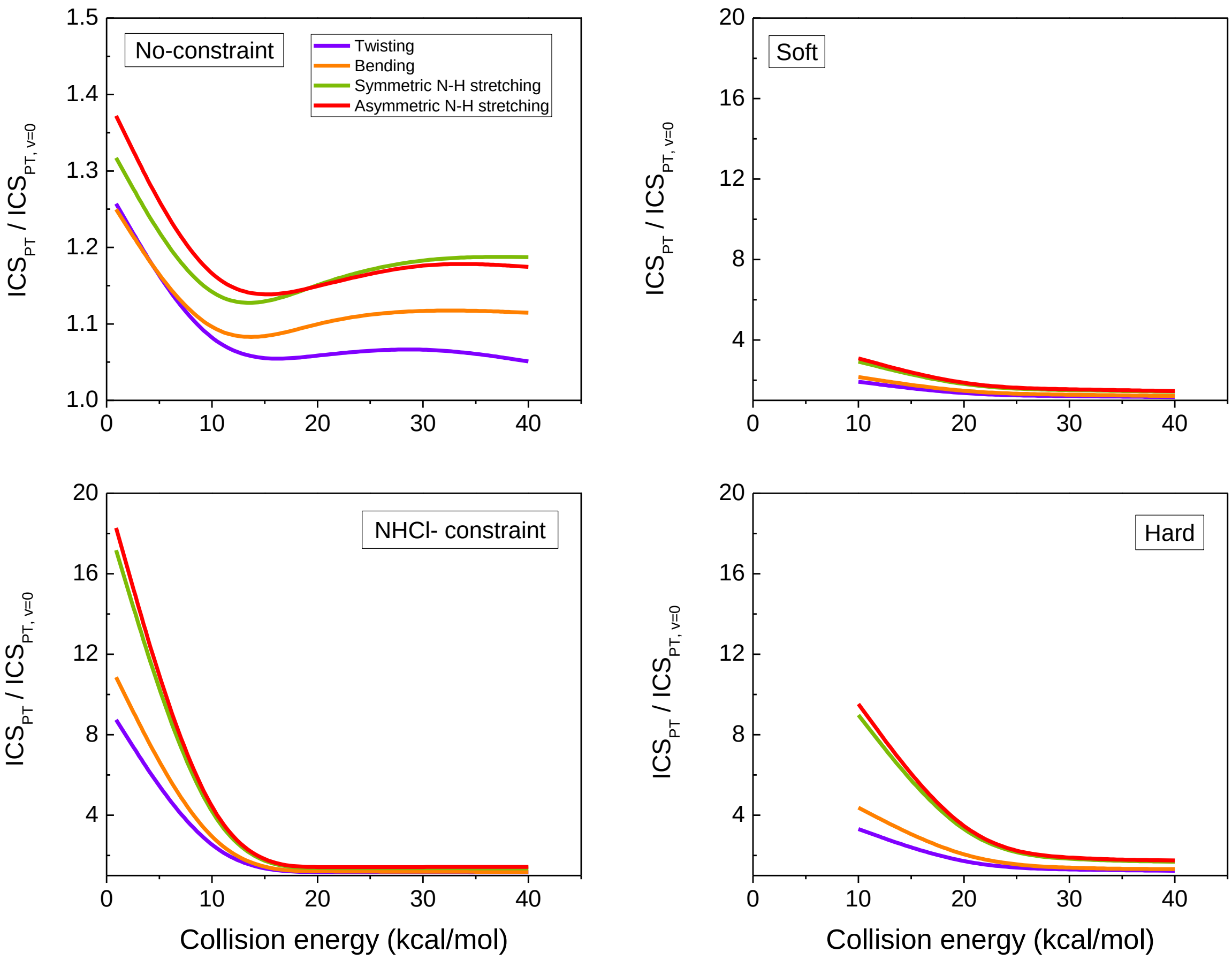

Figure S3. Ratios of the integral cross sections of the vibrationally excited and the non-excited (v = 0) proton-transfer channel of the F^- + NH_2Cl reaction in case of various excitations of the reactant vibrational normal modes (twisting, bending, symmetric and asymmetric N-H stretching) as a function of collision energy applying different zero-point-energy (ZPE) constraints: Soft - the sum of the vibrational energies of the products must be larger than the sum of the ZPEs, Hard - this restriction is set for each product separately, $NHCl^-$ constraint - the constraint is set only for $NHCl^-$.

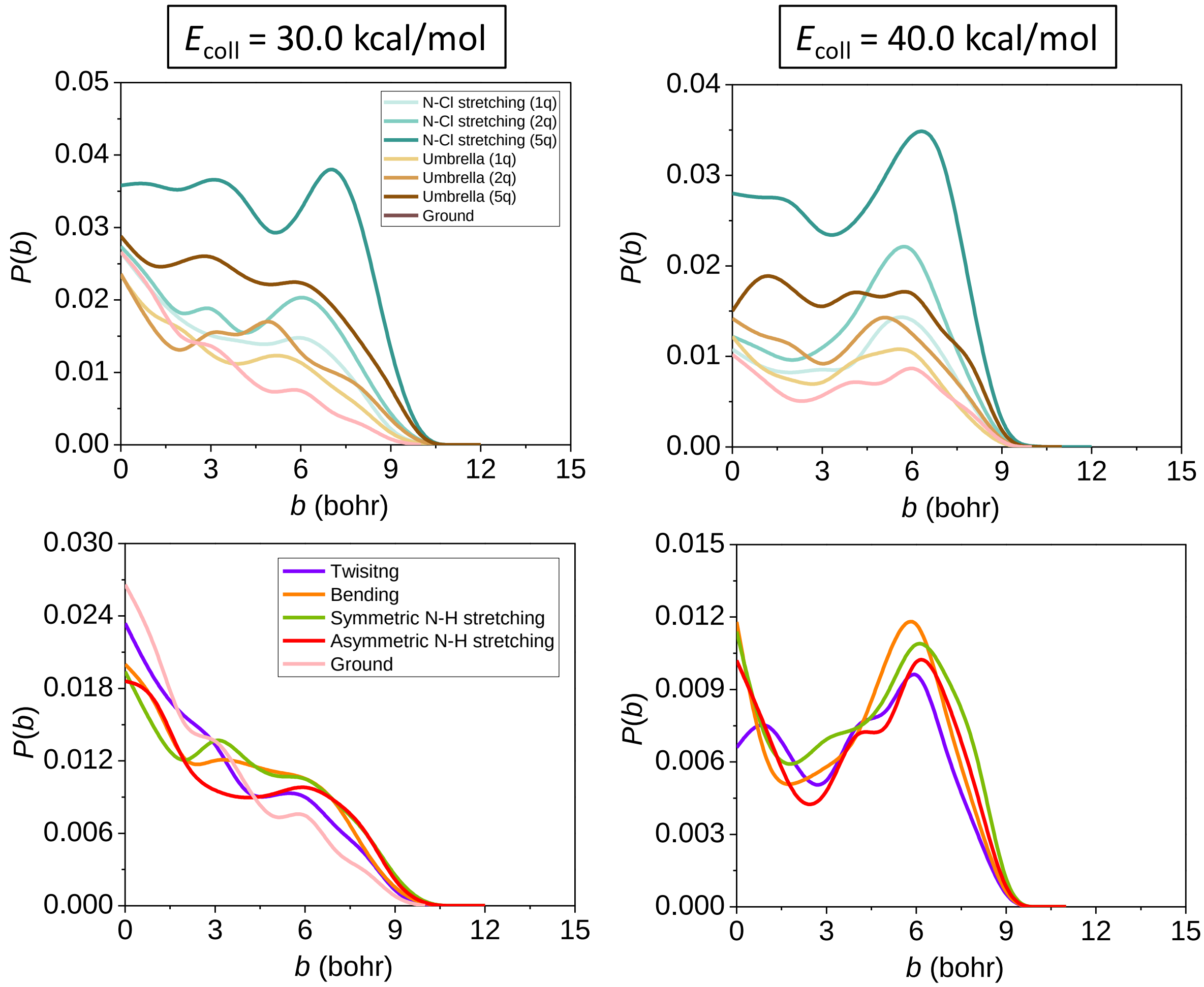

Figure S4. Opacity functions (reaction probabilities P as a function of the impact parameter b) of the $F^{-} + NH_2Cl \rightarrow Cl^{-} + NH_2F$ S_N2 reaction in case of various excitations of the reactant vibrational normal modes and for the unexcited reaction (Ground) at the two highest collision energies.

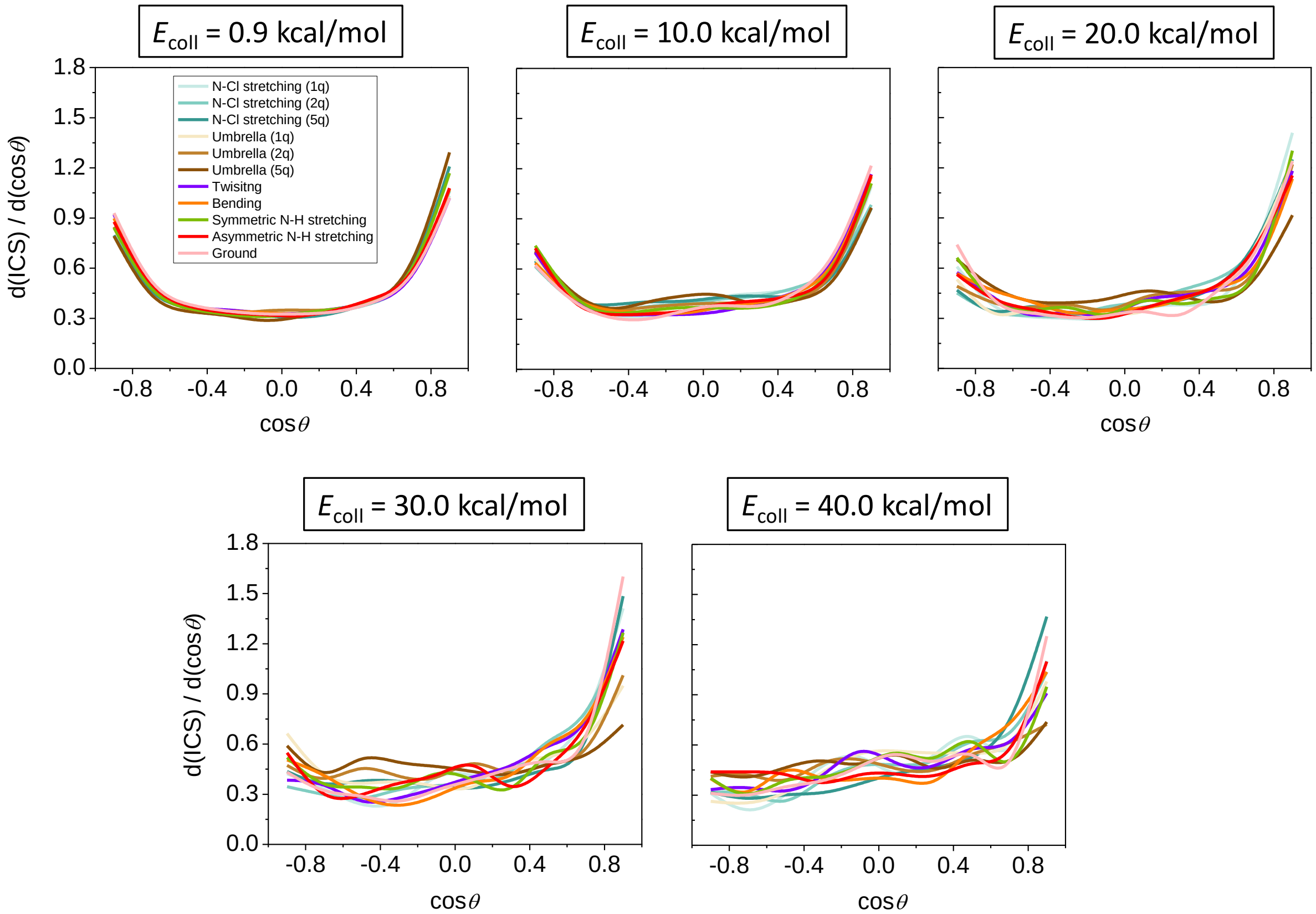

Figure S5. Scattering angle distributions of the F^- + NH_2Cl → Cl^- + NH_2F S_N2 reaction in case of various excitations of the reactant vibrational normal modes and for the unexcited reaction (Ground) at different collision energies.

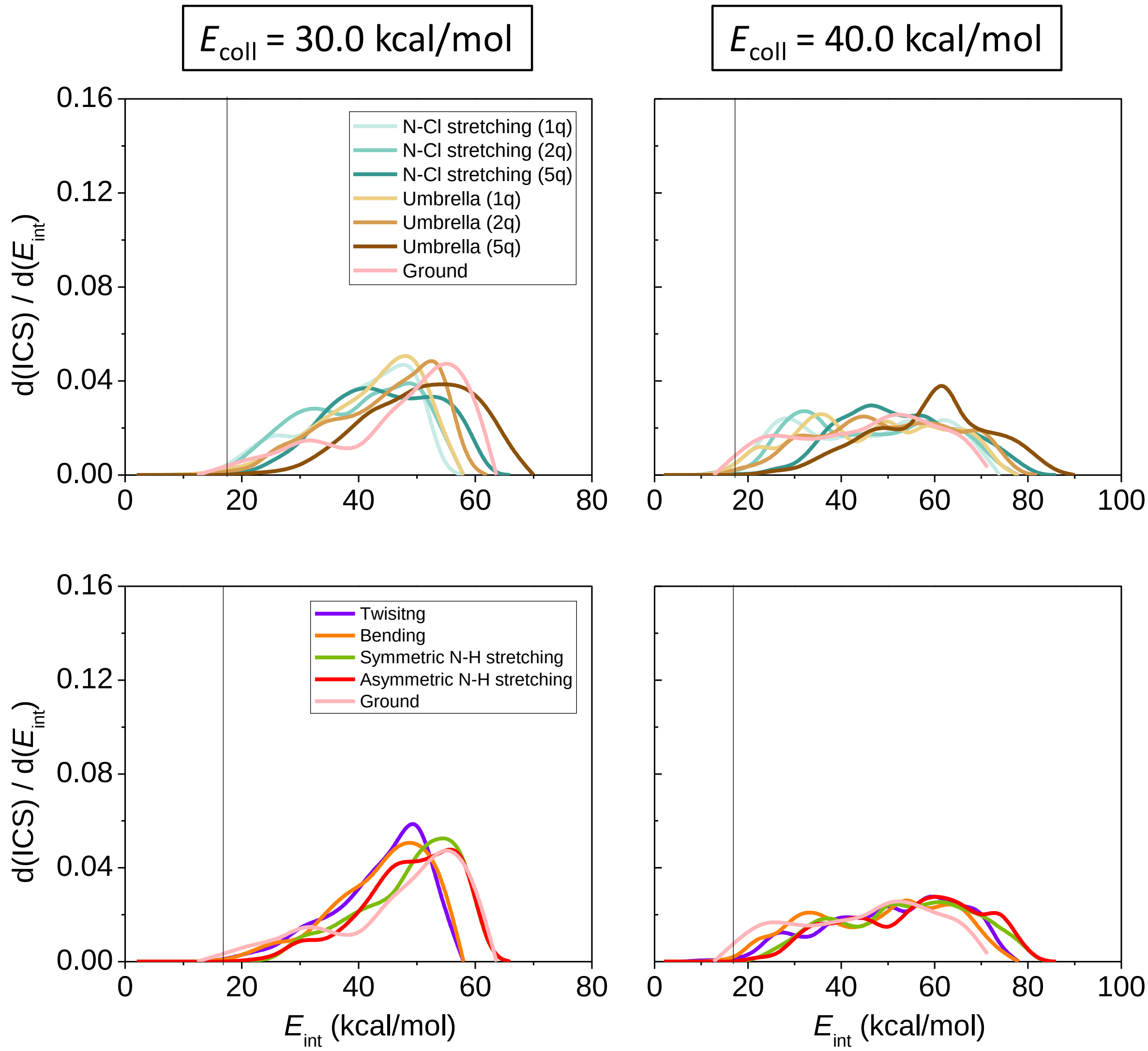

Figure S6. Internal energy distributions of the NH_2F product of the F^- + NH_2Cl → Cl^- + NH_2F S_N2 reaction in case of various excitations of the reactant vibrational normal modes and for the unexcited reaction (Ground) at the two highest collision energies. The vertical line denotes the ZPE of the NH_2F product.

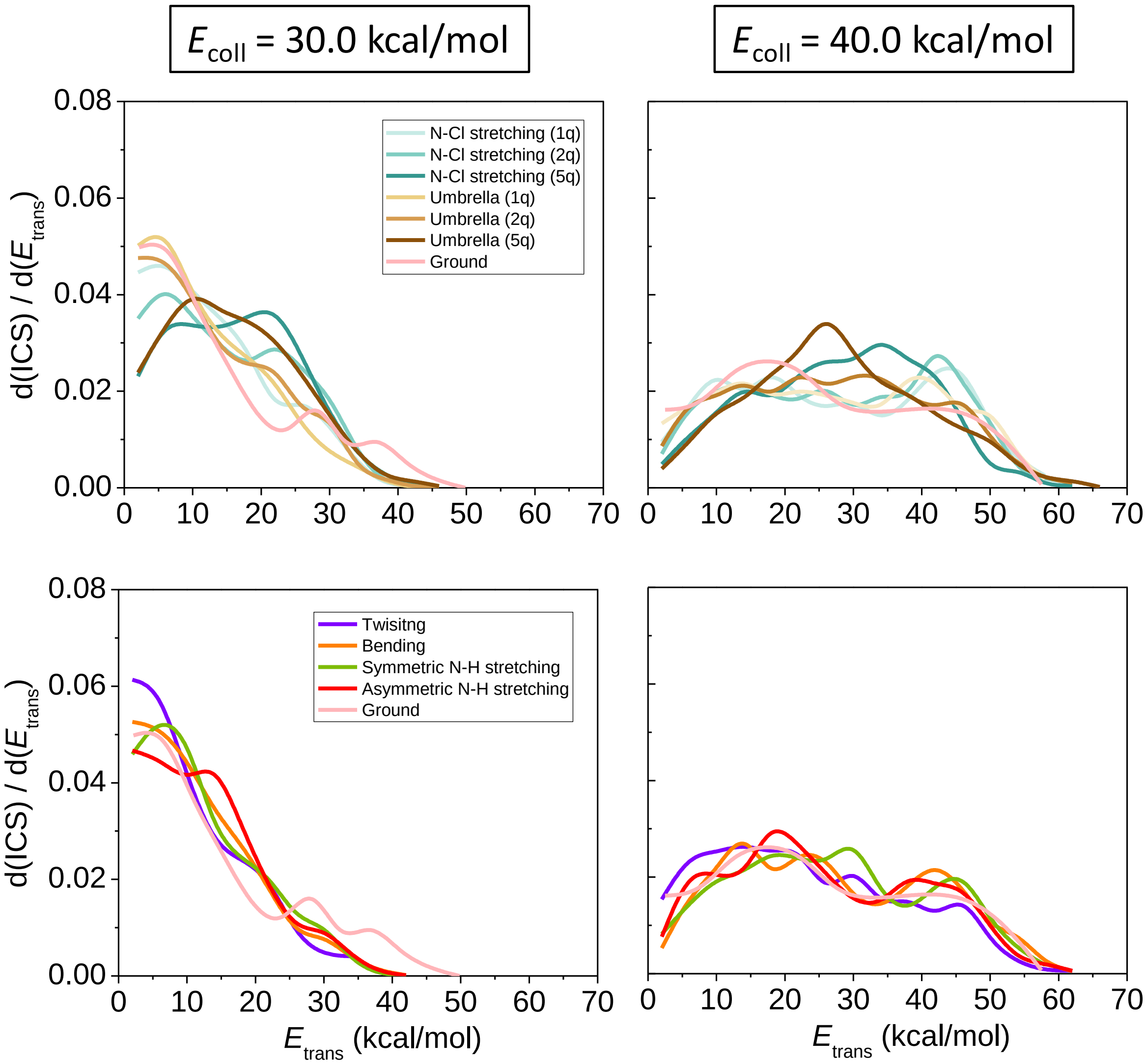

Figure S7. Relative translational energy distributions of the products of the $F^{-} + NH_2Cl \rightarrow Cl^{-} + NH_2F$ S_N2 reaction in case of various excitations of the reactant vibrational normal modes and for the unexcited reaction (Ground) at the two highest collision energies.

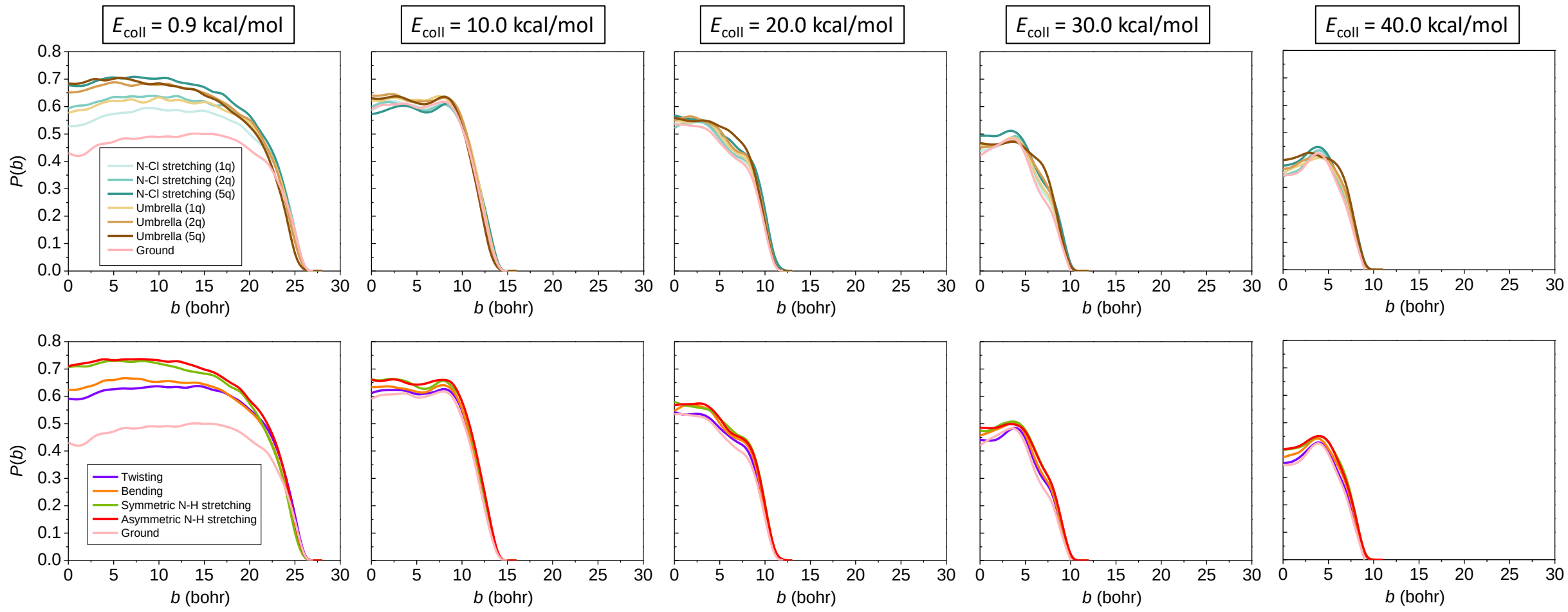

Figure S8. Opacity functions (reaction probabilities *P* as a function of the impact parameter *b*) of the $F^{-} + NH_2Cl \rightarrow NHCl^{-} + HF$ proton-transfer reaction in case of various excitations of the reactant vibrational normal modes and for the unexcited reaction (Ground) at different collision energies.

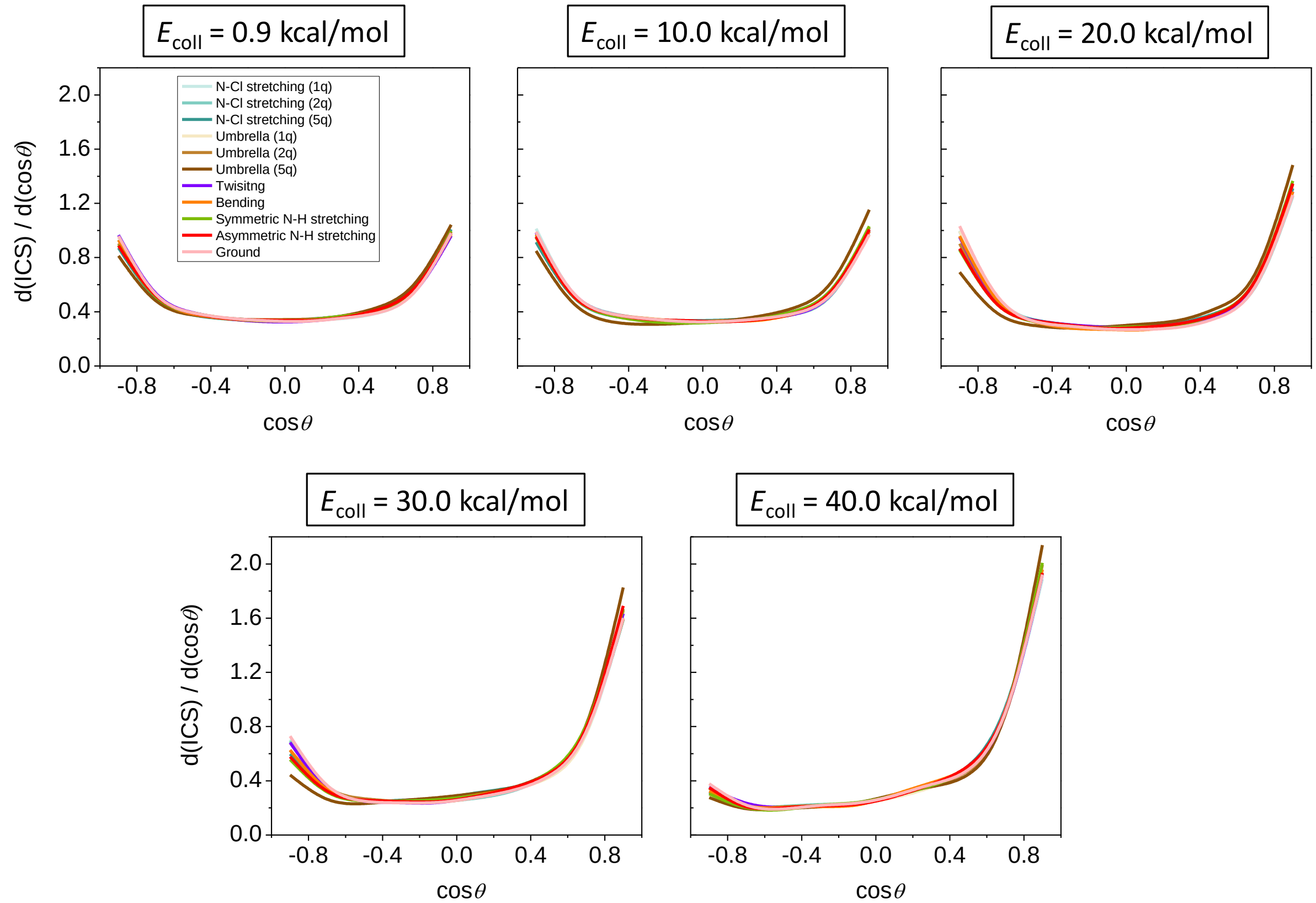

Figure S9. Scattering angle distributions of the F^{-} + NH_2Cl → $NHCl^{-}$ + HF proton-transfer reaction in case of various excitations of the reactant vibrational normal modes and for the unexcited reaction (Ground) at different collision energies.

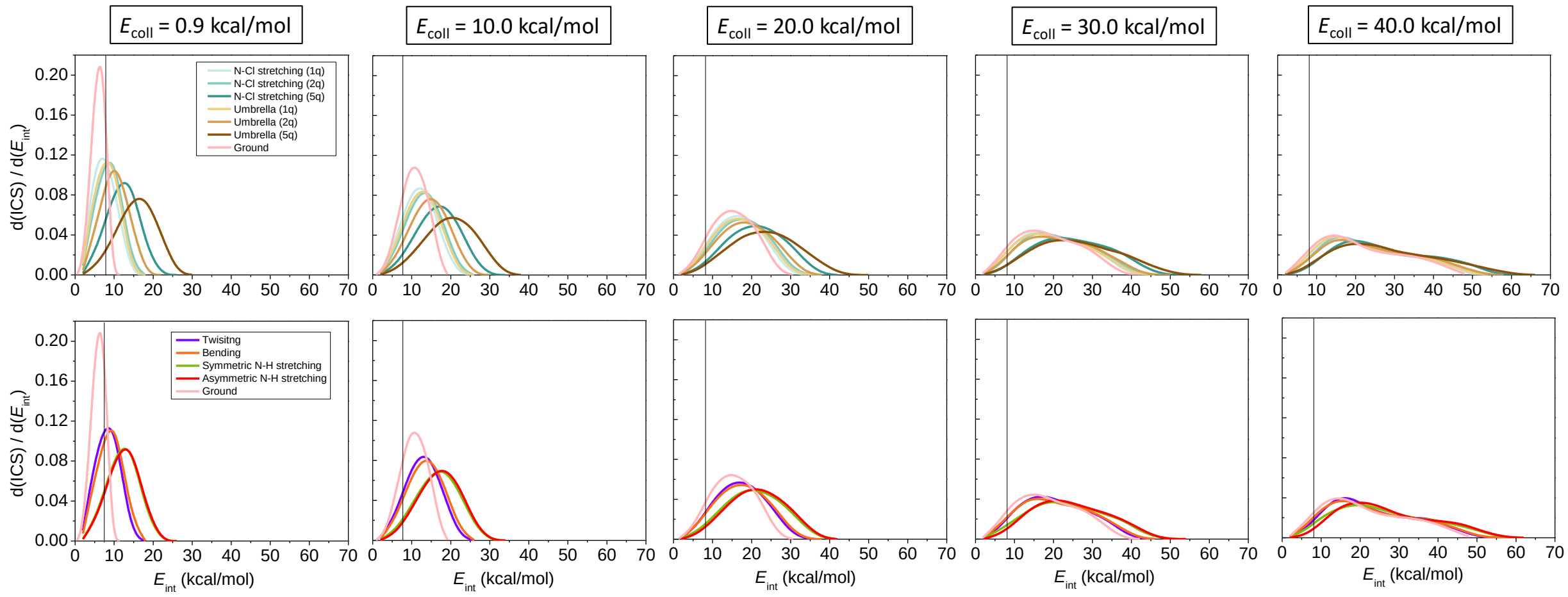

Figure S10. Internal energy distributions of the $NHCl^-$ product of the F^- + NH_2Cl → $NHCl^-$ + HF S_N2 reaction in case of various excitations of the reactant vibrational normal modes and for the unexcited reaction (Ground) at different collision energies. The vertical line denotes the ZPE of the $NHCl^-$ product.

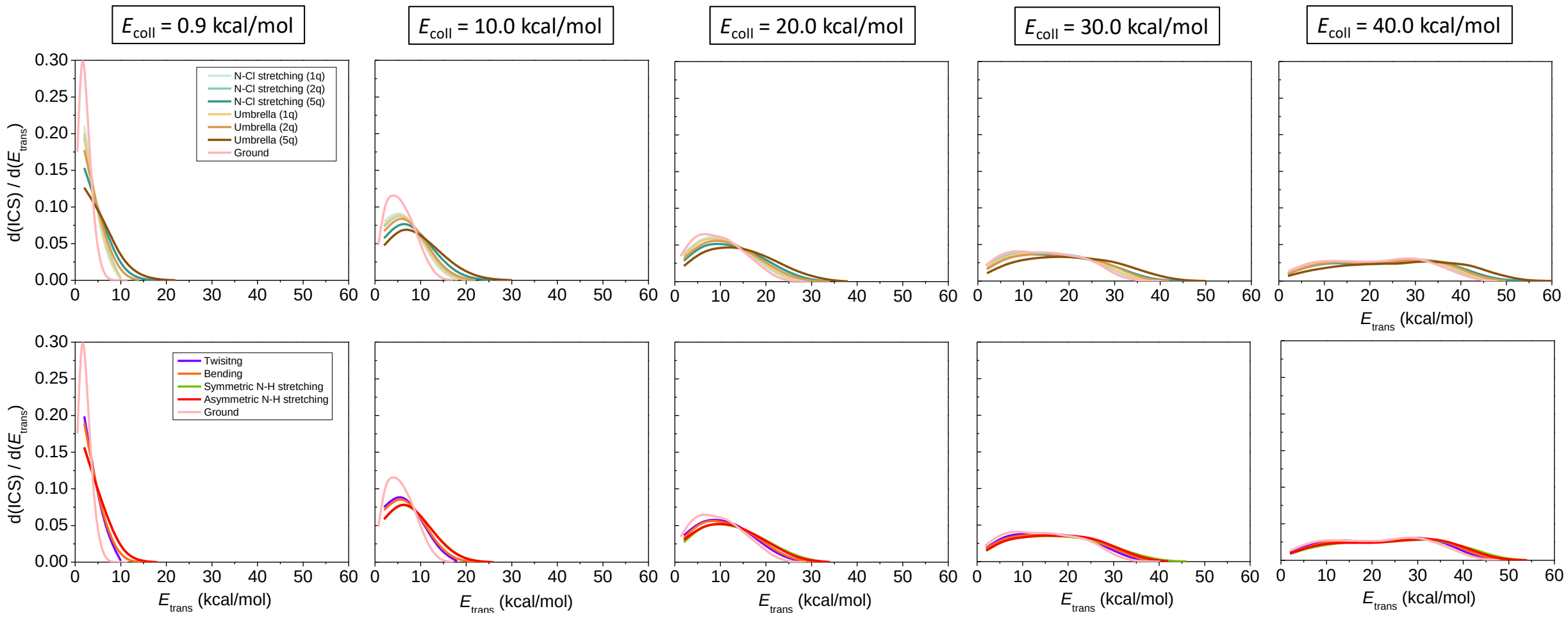

Figure S11. Relative translational energy distributions of the products of the $F^- + NH_2Cl \rightarrow NHCl^- + HF$ proton-transfer reaction in case of various excitations of the reactant vibrational normal modes and for the unexcited reaction (Ground) at different collision energies.
